# Neuropsychological and Balance Symptoms of Abused Women Who Have Experienced Intimate Partner Violence-Related Strangulation: A Feasibility and Acceptability Study

**DOI:** 10.1089/neur.2022.0047

**Published:** 2022-11-10

**Authors:** K. Jane Muir, Catherine Donahue, Donna K. Broshek, Jacob Resch, Nina Solenski, Kathryn Laughon

**Affiliations:** ^1^ National Clinician Scholars Program, University of Pennsylvania School of Nursing, Philadelphia, Pennsylvania, USA.; ^2^ School of Education and Human Development, University of Virginia, Charlottesville, Virginia, USA.; ^3^ School of Medicine, University of Virginia, Charlottesville, Virginia, USA.

**Keywords:** acquired brain injury, gender violence, intimate partner violence, non-lethal strangulation

## Abstract

Intimate partner violence (IPV) is a public health crisis that results in acute and long-term health consequences for women, including potential acquired brain injury from non-fatal strangulation. Despite existing evidence on the neuropsychological sequelae experienced by women after experiencing IPV-related assault, limited evidence-based treatment protocols exist for these women. This 14-month study sought to: 1) assess the feasibility and acceptability of recruiting women who experienced strangulation associated with IPV within 7 days of the event and retaining them throughout a 3-month follow-up period; and 2) examine preliminary data from neuropsychological, balance, and symptom assessments. Inclusion criteria were: reported strangulation by an intimate partner in the past 7 days, female, 18–60 years of age, English speaking, and able to consent. Neuropsychological, balance, and symptom assessments were administered at the first time point and again 3 months later. Participants also completed a standardized daily symptom inventory. Eight participants (73%) were recruited and completed daily inventories and the baseline assessment; 4 (36%) completed the baseline and 3-month assessments. Of the 4 participants who completed the 3-month assessment, none reported symptom resolution. Only balance returned to values consistent with normative values. Our results demonstrate the ability to recruit women who have experienced IPV-related strangulation during the post-acute phase of injury with less success retaining participants for a 90-day period for follow-up study. This pilot research protocol demonstrated the feasibility of recruitment from the emergency department and systematic evaluation of neuropsychological and functional symptoms in women who experienced strangulation in the context of IPV.

## Introduction

Intimate partner violence (IPV) is a public health crisis that results in acute and long-term physical and mental health consequences for women.^1–3^ IPV is defined as physical, emotional, and/or sexual abuse and controlling behaviors, which impacts ∼1 in 3 women in the United States in their lifetimes.^1^ Common areas of injury associated with IPV assault include the head, face, and neck.^2^ Further, 10% of U.S. women report non-lethal strangulation^1^ (pressure around the neck that impedes blood flow to the brain) resulting in periods of anoxia, hypoxia, urinary or fecal incontinence, facial or limb paralysis, dysphagia, and a range of other psychological, cognitive, neurological, and pathological outcomes.^4^ Valera and colleagues identified poor performance on long-term memory tests and high levels of depression among women who experienced strangulation-related alterations in consciousness.^5^ Acquired brain injury (ABI) resulting from assaults to the head and neck contributes to significant disability and cost burdens among women in the United States.^3–5^ It should be noted that although many studies of women who experience IPV refer to traumatic brain injury (TBI), ABI is a more accurate term when strangulation is involved given that this term includes injury attributable to anoxia or hypoxia.^1–3^ Approximately 25% of women who experience IPV experience repeat strangulations.^6–8^

Despite the burden of ABI for some women who experience strangulation, little prospective longitudinal data exist that capture the acute phase of injury through an extended period of follow-up after an IPV strangulation event. ^4^ Such data are critical given that it may allow clinicians to predict which patients may experience persisting symptoms and lead to early intervention and individualized care. Current recommendations for clinical evaluations do not fully assess the extent of strangulation-associated neuropsychological and functional deficits nor the symptom burden among women experiencing IPV.^8–10^ Understanding the neuropsychological and functional outcomes of women victimized by strangulation associated with IPV is necessary for future development of evidence-based protocols for the assessment and treatment of ABI in women experiencing IPV.

In this small feasibility study, we sought to develop and test a prospective, longitudinal protocol to characterize neuropsychological and functional changes in a sample of women recruited from the emergency department (ED) who had confirmed strangulation injury from an IPV event. More specifically, we aimed to explore and confirm the feasibility of our protocol to: 1) recruit and retain women who experienced strangulation associated with IPV within 7 days of the injury and throughout a 3-month follow-up period; and 2) characterize neuropsychological and functional assessment performance during the post-acute phase of injury and throughout a 3-month study period.

## Methods

### Setting

Participants were recruited from the ED of a level 1 trauma center in an academic medical hospital in the southeastern United States from November 2019 to January 2021. Study participants all received standard clinical treatment and referrals to supportive services, as determined by the clinical team in the ED. This study was approved by the institutional review board (IRB HSR #21773), and all participants provided consent before data collection.

### Participants

Women who experienced IPV strangulation were recruited to participate in this study over a 14-month study period (November 2020 through January 2021). Potential participants were identified by forensic ED nurses who then referred participants to the study coordinators by phone call if they met the study inclusion criteria. Flyers were also distributed throughout the community and in women's temporary shelters with a phone number that prospective participants could call if interested in participating in this study. Study inclusion criteria consisted of: 1) strangulation by an intimate partner in the past 7 days; 2) between the ages of 18 and 60 years; and 3) English speaking with the ability to provide written informed consent. After referral and determination of inclusion in our study, participants reviewed a consent form, were given the opportunity to ask questions, and then were consented for study participation by either the forensic ED registered nurses (RNs) or study coordinator. If the forensic ED RN consented the participant, they immediately informed the study coordinator to begin the study protocol. The study coordinator was an ED RN (K.J.M.) in the same ED where patients were evaluated.

### Procedures

**FIG. 1.**
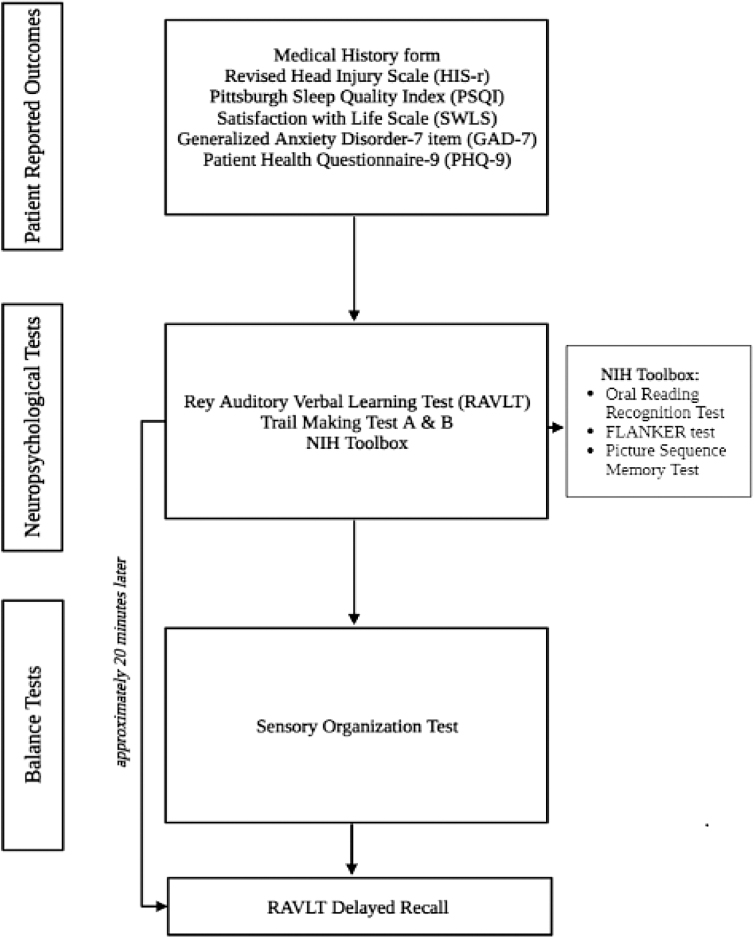
Testing sequence.

Participants completed an initial symptom inventory (Revised Head Injury Scale; HIS-r)^11^ and were scheduled for in-person testing in a separate research laboratory. The study coordinator scheduled participants for their first in-person testing session within 7 days of the strangulation event. The latter session served as the post-injury baseline session. The second in-person session occurred ∼3 months after session 1 and was scheduled by phone by the study coordinator. At both sessions, participants completed surveys, neuropsychological testing, and a balance assessment (see Fig. 1). Every day between the baseline and 3-month sessions, participants were asked to complete the HIS-r using their personal electronic device or a computer.^12^ Participants were given the option to complete all measures on paper if they did not have access to a personal electronic device or computer. The study coordinator reviewed instructions for the HIS-r with participants during study consent. Participants then completed the HIS-r survey daily until they reported being symptom free to the study coordinator. Additionally, the study coordinator (J.M.) called participants weekly to encourage completion of the HIS-r daily.

Security measures were discussed with participants to ensure their privacy. Participants were educated on the use of the escape key if taking the survey on a computer and/or the use of the lock screen on their personal electronic device. Participants were told how to clear their browser history, if desired, after taking the surveys. Although no participants opted to take the surveys on paper, that was an option for any participant who preferred paper-and-pencil instruments.

Participants were compensated $150 for attending each in-person session and $10 per week for participating in weekly phone calls. The study coordinator arranged participant transportation using a ride-sharing phone application on a mobile device. The study coordinator called the participants to ensure that the ride-sharing pickup was completed.

### Measures

#### Sociodemographics

Patients were administered a demographic questionnaire that consisted of participant age, identified sex, occupational status, educational level, and history of IPV-related injury.

#### Neuropsychological tests

##### National Institutes of Health Toolbox

To assess neuropsychological function, specific subtests of the National Institutes of Health (NIH) Toolbox were administered at each session. The NIH Toolbox was developed as an assessment tool for longitudinal, epidemiological, and intervention studies and provides measures of cognitive ability. More specifically, the NIH Toolbox consists of tablet-based measures of memory, attention, executive function, processing speed, language, and reading.^13^ Estimated baseline intellectual ability is evaluated by a word reading test.^14^ For our study, the following subtests were used.

##### Oral Reading Recognition Test

The Oral Reading Recognition Test^15^ evaluates familiarity with low-frequency vocabulary words by assessing the ability to read and pronounce letters and words as accurately as possible. Performance on word reading tests provides a proxy of general intellectual ability and access to and familiarity with written materials. The Oral Reading Recognition Test has been demonstrated to have strong test-retest reliability (*r* = 0.85) with a 15-month test-retest interval in healthy older adults and to have concurrent validity in comparison to the American National Adult Reading Test.^14^

##### Flanker Inhibitory Control and Attention (Flanker) Test

The Flanker test measures a participant's attention and inhibitory control.^15^ The Flanker test has been demonstrated to have moderate test-retest reliability (*r* = 0.62) over a 15-month test-retest interval and concurrent validity when compared to the Stroop Color Word Test.^14^

##### Picture Sequence Memory Test

The Picture Sequence Memory Test measures episodic memory in order to determine a participant's ability to acquire, store, and recall new information.^16^ The Picture Sequencing Memory test has been demonstrated to have acceptable test-retest reliability (*r* = 0.74) and concurrent validity when compared to the Rey Auditory Verbal Learning Test (RAVLT).^14^

#### Paper-and-pencil neuropsychological assessments

In addition to the NIH Toolbox Cognitive Battery, the RAVLT and the Trail Making Test (TMT) were administered by a trained examiner.

##### Rey Auditory Verbal Learning Test

The RAVLT is an assessment of multi-trial word list learning as well as immediate and delayed verbal recall memory. The RAVLT has been demonstrated to be reliable, with correlation coefficients ranging from 0.76 to 0.83.^17^

##### Trail Making Test A and B

The TMT consists of two parts. Part A measures attention, cognitive processing speed, visual scanning, and motor speed, whereas Part B also includes a speeded mental flexibility component.^18^ The TMT has been demonstrated to have moderate test-retest reliability (0.41–0.65) and demonstrated sensitivity in detecting cognitive change after concussion.^19,20^

### Balance assessment

#### Sensory Organization Test

The Sensory Organization Test (SOT) is a computerized measure of balance and is considered the “gold standard” of computerized posturography.^12^ For the current study, the SOT was administered on the Bertec Computerized Dynamic Posturography System (Bertec Corporation, Columbus, OH). During the SOT, participants completed three 20-sec trials for six conditions for a total of 18 trials. Each SOT condition presents a varying sensory challenge that measures somatosensory, visual, and vestibular input. The SOT yields an Equilibrium Score along with Somatosensory, Visual, and Vestibular sensory ratios. In the absence of pre-injury data, participant performance may be compared to normative values specific to age and height.^21^

In order to complete the SOT, all participants were first fitted with a safety harness to minimize the risk associated with potential falls. The importance of a safety harness to reduce the risk of falls was explained to participants, and a female research coordinator assisted with placement of the safety harness. Upon placement of the safety harness, participants entered the Bertec CDP and then received standardized instructions. After verbal acknowledgement of the instructions, 5-sec samples of each of the six SOT conditions were provided to familiarize participants to the test. After completion of the sample trials, participants were then administered the SOT.^22^ In order to reduce known practice effects with the SOT,^23^ the 18 trials were administered in a randomized order and the test took ∼15 min to complete. The SOT has been demonstrated to have variable test-retest reliability (0.35–0.79) based on varying sample compositions and acceptable validity with a sensitivity of 77.5% for detecting symptoms after concussion.^24–27^

### Patient-reported outcomes

#### Pittsburgh Sleep Quality Index

The Pittsburgh Sleep Quality Index (PSQI) is a 19-item questionnaire used to measure sleep quality during the preceding month.^28^ A global score >5 is indicative of poor sleep quality.

#### Revised Head Injury Scale

The HIS-r^12^ consists of 22 concussion-related symptoms measuring symptom quantity, duration, and severity over a 24-h period. The HIS-r survey was delivered to participants by a Qualtrics link they could access on their personal devices or on a laptop computer. To complete the HIS-r, participants first endorsed whether they had experienced 1 of 22 symptoms during the previous 24 h. If a symptom was endorsed, participants then rated the symptom on scales of duration and severity. For duration, each endorsed symptom was rated on a Likert scale ranging from 1 (“briefly”; i.e., 15 min) to 6 (“always”). For severity, each endorsed symptom was rated on a Likert scale ranging from 0 (“not severe”) to 6 (“as severe as possible”). In addition to the total number of symptoms endorsed (ranging from 0 to 22), total symptom duration and total symptom severity scores were calculated by summing the respective individual duration and severity scores for each symptom, resulting in a total score ranging from 0 to 132 for each respective outcome. The HIS-r has been demonstrated to have high sensitivity and specificity in collegiate athletes diagnosed with a sport concussion, but has not been previously used in the population studied here.^25^

#### Satisfaction With Life Scale

The Satisfaction With Life Scale (SWLS) is a five-item scale designed to assess an individual's global judgement of life satisfaction.^29^

#### Generalized Anxiety Disorder 7-Item Scale

The Generalized Anxiety Disorder 7-Item (GAD-7) scale is a seven-item scale that assesses the presence and duration of anxiety symptoms.^30^ The resulting total score ranges from 0 to 21, indicating levels of anxiety as minimal (score of 0–4), mild (5–9), moderate (10–14), and severe (15–21).

#### Patient Health Questionnaire-9

The Patient Health Questionnaire-9 (PHQ-9) is a nine-item scale that assesses the presence and duration of depressive symptomatology.^30^ The resulting score ranges from 0 to 27, indicating levels of depression as minimal (score of 0–4), mild depression (5–9), moderate depression (10–14), moderately severe depression (15–19), or severe depression (20–27).

### COVID-19 note

Of note, recruitment and testing for the study was paused from March 2020 until July 2020 because of COVID-19. Per institutional policy, participants who were already consented for the study were able to continue in the study; however, any scheduled in-person testing was canceled. Recruitment of new participants into the study was prohibited until July 2020. When testing was again permitted with COVID-19 precautions, the study team updated the study protocol to protect participant health and safety. Specifically, the study team created and simulated a trial run of completing the in-person testing using personal protective equipment, COVID-19 symptom tracking, and alternative entrances to the laboratory. Participants continued to receive weekly HIS-r phone calls from the study coordinator during the COVID-19 shutdown.

### Protocol

After enrollment, participants were provided transportation to the university research laboratory by a ride-share service. During each session, two investigators were present with at least one investigator being female. Upon arrival, participants were administered the previously described measures in the following order: patient-reported outcomes (medical history form, HIS-r, PSQI, SWLS, GAD-7, and PHQ-9); neuropsychological tests (RAVLT, TMT, and NIH Toolbox); and balance assessment (SOT). The RAVLT long-delay free recall was administered after the SOT because of the required 20-min interval after the short-delay recall (see Fig. 1). Each session lasted ∼60 min.

### Statistical analysis

All data were deidentified and stored in a locked cabinet in the principal investigator's office to protect participant information. Data analysis included frequency distributions of categorical variables and descriptive statistics (means and standard deviations) for test scores. All data were analyzed in Statistical Package for Social Sciences (v.28; IBM Corp., Armonk, NY).^31^

## Results

Over the 14-month study period, inclusive of the limitations imposed by COVID-19, a total of 11 participants (*n* = 11) were enrolled. Of the participants enrolled, 3 (27.3%) completed informed consent forms but were unable to schedule the baseline assessment within 7 days of their injury. The remaining 8 participants (72.7%) completed their post-injury baseline session. Of those participants, 4 (36%) completed the 3-month session. The 4 participants who did not complete the 3-month session were not eligible to do so because of the COVID-19 shutdown. Participant demographics are listed in Table 1. Data are reported on the 8 participants who completed the post-injury baseline session in Table 2. All participants completed the HIS-r using an electronic device between the post-injury baseline and 3-month sessions. Among the participants able to attend in-person sessions, all were successfully able to use the provided ride-share services for transportation without incident.

**Table 1.** Participant Demographics, *n* = 8

Variable	Value
Age, years (mean ± SD)	27.00 ± 12.80
Race (counts)	
White	7
Asian/Pacific Islander	1
Education level (counts)	
Grade 11	1
High school graduate	1
Associates degree	4
Bachelor's degree	2
Time post-event, days (mean ± SD)	1.90 ± 2.35
Symptom severity at enrollment (mean ± SD)	58.30 ± 21.99

^*^ Demographic data at time of enrollment.

SD, standard deviation

**Table 2.** Means and Standard Deviations of Participant Results at Session 1 and Session 2

	Post-injury baseline session (Avg [SD]) (*N* = 8)	Three-month (Avg ± SD) (*N* = 4)
RAVLT A1 A2 A3 A4 A5 B1 A6 A6D	46.7 (2.87) 5.1 (1.54) 7.5 (1.50) 9.1 (2.03) 8.3 (3.43) 11.6 (1.93) 4.9 (1.45) 8.4 (2.91) 7.6 (2.43)	45.0 (1.63) 4.5 (1.12) 7.0 (1.41) 9.5 (1.12) 10.0 (3.24) 10.5 (3.35) 5.0 (1.22) 8.3 (3.49) 7.8 (3.11)
Trail Making A (sec)	35.4 (12.11)	36.5 (15.05)
Trail Making B (sec)	73.4 (32.96)	109.04 (39.67)
PSQI	25.0 (9.09)	23.3 (8.29)
GAD-7	16.5 (3.04)	15.8 (3.49)
SWL	12.6 (4.69)	9.5 (3.91)
PHQ-9	18.0 (4.5)	17.5 (6.22)
HIS-r No. of symptoms Symptom severity Symptom duration	13.3 (2.81) 53.5 (18.64) 55.2 (19.05)	(note: *n* = 8) 13.5 (2.70) 52.5 (16.38) 56.0 (14.04)
NIH Oral Reader	101.1 (19.30)	104 (14)
NIH Flanker Inhibitory	8.5 (1.68)	7 (1.33)
NIH Picture Sequence	532.0 (92.1)	525.1 (139.29)

RAVLT, Rey Auditory Verbal Learning Test; PSQI, Pittsburgh Sleep Quality Index; GAD-7, 7-item Generalized Anxiety Disorder; SWL, Satisfaction With Life scale; PHQ-9, 9-item Patient Health Questionnaire; HIS-r, Revised Head Injury Scale; SD, standard deviation.

### Revised Head Injury Scale

**FIG. 2.**
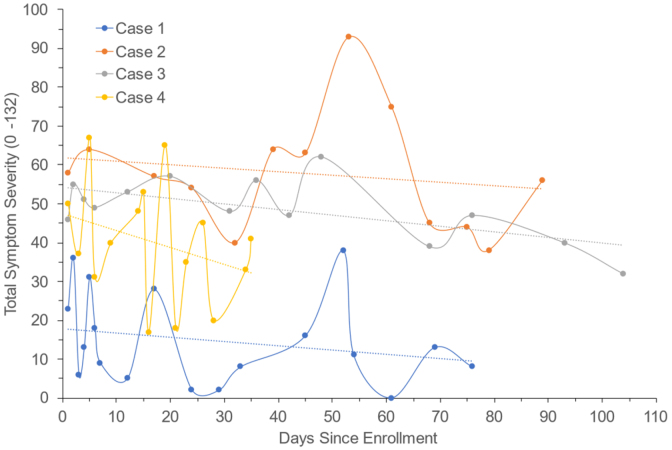
Revised Head Injury Scale scores over time.

Among all study participants (*n* = 8), the average total HIS-r symptom severity score obtained at time of study consent (i.e., within 72 h of injury) was 58.30 ± 22.00 (out of 132). At the post-injury baseline assessment (∼5 days after injury), participants reported, on average, 13.20 ± 2.81 symptoms (out of 22), with an average symptom severity of 53.50 ± 18.64 and average symptom duration of 55.20 ± 19.05 (out of 132) during the previous 24 h. At the 3-month follow-up (session 2), participants (*n* = 4) reported, on average, 13.50 ± 2.70 symptoms, with an average symptom severity of 52.50 ± 16.38 and symptom duration of 56.00 ± 14.04 (see Fig. 2). Participants were asked to fill out a daily HIS-r until their symptoms remitted. None of the participants achieved symptom resolution by session 2 (3 months). Participants reported high levels of sleep disturbance, mean anxiety scores consistent with severe anxiety, and depressive symptoms consistent with moderately severe depression at both time points (see Table 2).

### Other study measures

Descriptive statistics on NIH Toolbox tablet-based measures, individually administered neuropsychological measures, and other patient-reported measures are noted in Table 2. At the post-injury baseline session, equilibrium (composite) balance (SOT) scores were 78.60 ± 17.25, compared to the post-test (86.60 ± 4.07), suggestive of a 10% improvement for the SOT composite equilibrium score (see Table 3). Mean SOT equilibrium score was below the normative values during the post-injury baseline session, whereas mean SOT equilibrium score was consistent with normative values for height and age at the 3-month session. Participants reported symptoms on the PHQ-9 consistent with severe depression (baseline, 25,00 ± 9.09; 3-month, 23.30 ± 8.29) and scores on the GAD-7 consistent with severe anxiety (baseline, 16.50 ± 3.04; session 2, 15.80 ± 3.49).

**Table 3.** Means and Standard Deviations for the Sensory Organization Test for Participants Completing Visit 1 and Visit 2 (*n* = 4)

Subscale	Normative values^11^	Session 1 (mean ± SD)	Session 2 (mean ± SD)
Composite	79.8 (5.63)	78.60 ± 17.25	86.60 ± 4.07
Somatosensory ratio	98.0 (0.05)	95.30 ± 4.57	96.50 ± 1.73
Visual ratio	87.7 (8.00)	89.80 ± 11.87	93.30 ± 4.19
Vestibular ratio	73.6 (11.1)	68.30 ± 33.69	85.80 ± 3.86

SD, standard deviation.

## Discussion

The purpose of this study was to explore and confirm the feasibility of a research protocol to characterize the clinical presentation of patients injured by IPV with strangulation. Of our 11 consented participants, 72% were evaluated within 5 days of their injury using a multi-dimensional assessment. Of the 8 remaining participants, each subject completed a digitized HIS-r daily throughout the study duration. Only 4 (*n* = 4) participants completed the follow-up session, which occurred 3 months after the baseline assessment, but it should be noted that the inherent challenges of follow-up with this population were further complicated by the COVID-19 pandemic. Although statistical analyses were limited because of small sample size, these findings showed an improvement in mean equilibrium SOT scores from below-normative to normative levels between the two testing sessions. In sum, the obtained results support the feasibility of this research protocol during the post-acute phase of injury, despite the inherent challenges when conducting a prospective, longitudinal study of women who have experienced strangulation in the context of IPV.

Our protocol successfully captured initial symptom assessment (HIS-r) within 72 h of ED evaluation, at which point participants reported a high total symptom severity.^32^ Current research demonstrates^33,34^ that a higher symptom severity immediately after concussion predicts a prolonged recovery. In our sample of women who experienced IPV-related strangulation, none of our participants reported a low symptom severity or reported as symptom free during any point in our study. Though the findings of this small feasibility study must be interpreted with caution, results suggest that assessment of ABI symptoms as part of the IPV assessment protocol in the ED is critical to understanding the nature and severity of the injury.

Anxiety and mood disorders are also associated with prolonged recovery post-concussion.^35,36^ Our participants reported high scores on measures of depression and anxiety using validated clinical measures, which is consistent with existing evidence on strangulation injuries among abused women.^4^ It is important to acknowledge this within the context of evidence suggesting that multiple ABIs are positively correlated with post-injury depression and anxiety symptoms.^37,38^ Whereas symptoms of depression and anxiety may be etiologically related to ABI, these symptoms almost assuredly also reflect the psychological sequelae of abuse or repeated abuse (i.e., repeated abuse-related events) within the context of an intimate partner relationship. Clarification of the relationship between these variables is beyond the scope of the current study and requires future research.

In this study, we successfully recruited participants and completed a symptom inventory within 72 h of injury. Related literature has documented time to recruitment of women who experienced IPV of several weeks or months after assault.^39,40^ Our study findings do demonstrate that use of the HIS-r scale in an electronic format (such as a smartphone) was acceptable and safe for participants. Of the participants who completed the baseline assessment, each participant was able to successfully access the HIS-r survey from their smartphones and complete for ∼1 month of survey data over the study period. Finally, all participants who attended in-person sessions were able to successfully use the ride-share transportation set up for them as part of the research protocol.

Our findings are consistent with past studies assessing ABI^39–41^ among IPV survivors in that our participants reported a high frequency of persisting psychological symptoms (i.e., anxiety, depression, and sleep disturbances) after strangulation events. Although this study was not designed to diagnose ABI among participants, the elevated symptom severity scores on the HIS-r at study enrollment with persisting high symptom severity throughout the study period among all study participants were consistent with symptoms experienced by persons with diagnosed ABI.^32–34^ Future research is needed to better characterize the cognitive, physiological, and psychological impact of IPV with strangulation to inform evidence-based assessment and treatment protocols during the acute and post-acute time points along with a longitudinal design.

### Future studies

Our study established the feasibility of a research protocol that can be replicated in future studies. Our findings suggest the need to develop and test: 1) validated, comprehensive clinical neuroimaging protocols for IPV survivors experiencing head injury/strangulation events; and 2) establish clinical protocols for follow-up assessment and treatment for IPV survivors for associated cognitive, physiological, and psychological symptoms. Our study also underscores the difficulty in recruiting this highly vulnerable population for longitudinal research. Future studies will need to account for the logistical difficulties of enrolling women who have recently experienced non-lethal strangulation beyond a 7-day period. These data are critical to understand the trajectory of symptoms after IPV-related events. More comprehensive data are necessary to understand the clinical presentation for women who experienced strangulation during IPV and inform recommendations for evaluation, treatment, follow-up care, and community resources and provide a greater understanding of the unique challenges that might be faced by these women. Future development of this protocol for evaluating symptoms after strangulation-related IPV could form the foundation of a concerted national effort to standardize the initial clinical assessment in the ED and make evidenced-based recommendations for neuroimaging, tailored clinical care, and access to resources for those with ABI.

### Limitations

Our study had several limitations. This study was conducted during the COVID-19 pandemic, which resulted in a pause in participant recruitment and loss of recruited participants for the 3-month assessment. Most study participants were detrimentally impacted by the COVID-19 pandemic through lost employment, unstable housing, and reduced transportation options. Some participants experienced challenges maintaining study participation when their financial situation limited their use of cell-phone data, impacting their ability to complete the daily HIS-r survey. We were able to mitigate this through planned weekly phone calls by the study coordinator and did not lose contact with any participants. Future studies should anticipate and address social and financial barriers that could limit study completion among this vulnerable population.

## Conclusion

This study examined the feasibility and acceptability of a pilot protocol evaluating neuropsychological and physiological symptoms among abused women who experienced IPV strangulation. Despite the impact of COVID-19 on recruitment, the majority of recruited participants completed their initial assessment. Four (*n* = 4) participants completed both in-person assessment sessions separated by ∼3 months. Additionally, the use of a digitized symptom inventory allowed for all participants who completed their first assessment to contribute symptom data for ∼1 month. Participants reported increased symptom severity on a TBI symptom scale without symptom resolution during the study period. Our findings provide useful data for the development of a future, larger study to examine symptoms of ABI and psychological distress among a larger sample of abused women. Future research is critical to develop clinical protocols for the evaluation of ABI in women presenting to the ED after IPV that includes strangulation and/or TBI.

## Transparency, Rigor, and Reproducibility

The analysis plan was not formally pre-registered, but the team members primarily responsible for the analysis (C.D., J.R., and K.L.) certify that the analysis was pre-certified. Regarding sample-size calculation: After consultation with a statistician, it was determined that the lack of previous data from this population precludes a meaningful power calculation. Study data were collected between August 2019 and February 2021 and were analyzed in SPSS software (see the Methods).

## Abbreviations Used

ABI: acquired brain injury
ED: emergency department
GAD-7: Generalized Anxiety Disorder 7-Item scale
HIS-r: Revised Head Injury Scale
IPV: intimate partner violence
NIH: National Institutes of Health
PHQ-9: Patient Health Questionnaire-9
PSQI: Pittsburgh Sleep Quality Index
RAVLT: Rey Auditory Verbal Learning Test
RNs: registered nurses
SOT: Sensory Organization Test
SWLS: Satisfaction With Life Scale
TBI: traumatic brain injury
TMT: Trail Making Test
